# Progress in Medicine

**Published:** 1898-07-09

**Authors:** 


### Progress in Medicine.

### DISEASES OF CHILDREN.

**Multiple Sclerosis.**—Three cases of this rare affection in childhood have been recorded by Dr. Leopold Streglitz,[1] along with some valuable remarks on its differential diagnosis from other forms of nervous disease met with in early life. His patients were aged 9, 11, and 15 years respectively. The leading symptoms are the intention tremor, nystagmus, and bradylalia, and others are rigidity of the limbs, increased knee-jerks, and ankleclonus, a cerebellar ataxoparaplegic gait, and ocular paralysis. The diseases which are most likely to be mistaken for this affection are infantile cerebral palsy, congenital or acquired, syphilis of the cerebro-spinal axis, Friedreich's disease, and hereditary cerebellar ataxy. As regards the etiology, the author is inclined to lay equal stress on two influences—firstly, a previous attack of infectious disease, and secondly, a neuropathic taint. The prognosis is not so unfavourable as in the case of adults, several authenticated cases of recovery having taken place, and in others there has been a remission of symptoms for prolonged periods.

The value of lumbar puncture was discussed at the International Medical Congress in Moscow.[2] Von Ranke (Munich) thought the proceeding harmless, but the therapeutic value as tested in one hundred collected cases was nil. While still maintaining an open mind as regards its diagnostic value, he pointed out that in tuberculous cases it was frequently impossible to detect the bacillus in the spinal fluid. Monti (Vienna) held very similar views from his experience in cases of basilar tuberculous meningitis, epidemic cerebro-spinal meningitis, and acute hydrocephalus. Raczynski (Cracow) had tested this method of treatment in twenty-one cases of congenital or acquired hydrocephalus, in which the cause was unknown, and in five cases following cerebro-spinal meningitis. He had noted temporary reduction in the size of the skull for a few days, but the condition soon recurred, and not a single case was cured.

**Tetany.**—Dr. Floyd Crandall[3] states that the onset of an attack is usually sudden and without any warning. The spasms may be limited to the arms and legs, and more rarely the muscles of the face, neck, and trunk may be affected. Glottic spasm is a frequent accompaniment. The other diagnostic points are that the temperature is normal, there is no loss of consciousness, and the knee-jerks and cutaneous reflexes are exaggerated.

**Vesical Calculus.**—In the Lettsomian lectures on the affections of the urinary apparatus in children, Mr. John Morgan[4] lays down the following conclusions regarding the treatment of vesical calculus. In the cases of boys and girls stones of moderate size should be dealt with by litholapaxy. Stones composed of oxalate of lime, of such size as not to be readily grasped between the blades of a lithotrite, should be removed by the lateral operation in the case of boys. The suprapubic operation should be reserved for stones of very large size or inconvenient shape in boys or girls, or cases of calculus embedded in a saccule of the bladder, or impacted in the mouth of a ureter.

**Imbecility.**—In an interesting paper Dr. John Thomson[5] discusses the diagnosis and prognosis of certain forms of imbecility in infants, those, namely, associated with some bodily deformity. As regards microcephalic children the prognosis is not good either as regards life or mental development, and the evidence as regards improvement from operative treatment is still wanting. Hydrocephalic children have a surprising amount of intellect, considering the small amount of brain tissue, and those who survive are usually gentle and amiable in disposition, and capable of being educated up to a certain point. In cerebral infantile paralysis the degree of mental impairment is very variable. Even although mental instability and emotional weakness have been marked in infancy, the survivors are capable of a considerable amount of education. As regards Mongolian or Kalmuc imbeciles, it may be foretold that they will always be of a very low grade mentally, but they will be cleanly in their habits, and amiable and affectionate in their disposition. Dr. Clement Dukes[6] suggests that in some cases of "backward children"—those in whom mental and physical development seem delayed without any obvious cause—the administration of thyroid extract may sometimes be found serviceable. Such was his experience in the case of a girl of thirteen, who was stunted and anæmic. Under treatment by one 5-grain thyroid tabloid once a day, and later twice a day, she became a different child, losing her pallor, and gaining in height and intelligence.

**Paroxysmal Hyper-acidity.**—This condition in children has been found by Dr. Soltau Fenwick[7] to simulate very closely attacks of migraine. The symptoms are headache, commencing in the frontal or occipital region, but soon becoming diffused over the scalp, burning pain in the epigastrium, screaming of the meningeal type, flatulence, nausea, giddiness, and finally vomiting. The attack may rapidly pass off, but in other cases it is prolonged for two or three days. The affection is to be distinguished from ordinary gastric catarrh by the much greater severity of the symptoms. During an attack the best treatment is hot water to drink, or an emetic, or a full dose of antipyrin or phenacetin, and in preventing the recurrence of the disease the writer has found most benefit from bromide of potassium combined with liquor potassæ.

**Ophthalmia Neonatorum** has been successfully treated by Dr. A. F. Poukalow[8] with insufflations of calomel. After the eyes have been carefully bathed with a 2 per cent. solution of boracic acid, the lids are everted and calomel is blown over them and the corneæ, the more freely the better. The cases in which this treatment was employed were those of gonorrhœal origin.

**The Surgical Treatment of Microcephaly.**—This subject was brought before the Royal Medical and Chirurgical Society in a paper by Mr. Joseph Griffiths.[9] He had operated in the case of a boy aged sixteen months, and some improvement followed a linear craniectomy on the right side. A second operation on the opposite side was unfortunately followed by septic meningitis and death. He divided cases of this affection into two classes: (*a*) those with small, ill-filled, but not deformed skulls, and (*b*) those with small and deformed skulls. In the former class the brain is at fault, being arrested in development, and in the latter class there is, in addition, evident disease of the cortex, which determines the shape of the skull. A condition in which the skull is prematurely synostosed, and the enclosed brain normal, is purely hypothetical. Operation, therefore, did not afford much prospect of cure, but it might lead to amelioration of the symptoms in those cases in which there were symptoms of irritation of the motor cortical centres.

**The Surgical Treatment of Hydrocephalus.**—In a communication to the Clinical Society of London, Dr. G. A. Sutherland and Mr. Watson Cheyne[10] related a case of chronic hydrocephalus treated by intracranial drainage. Proceeding on the assumption that there was some mechanical interference with the outflow of fluid from the lateral ventricles *per vias naturales*, they tapped the ventricle through the cortex, and allowed the fluid to flow into the subarachnoid space, whose powers of absorption are very great. One end of a catgut drain was pushed into the ventricle, and the other end was passed under the dura mater, the wound in the dura being then tightly stitched up. No bad symptoms followed, and the steady shrinking in the size of the head which followed showed that the fluid was being drained in the manner anticipated. Three months later symptoms of basilar meningitis appeared, from which the child ultimately died. They thought that in early cases of hydrocephalus, before the brain tissue was destroyed by pressure, this method of sub-dural drainage gave a reasonable prospect of cure.

[1] Amer. Jour. Med. Sci., Feb. 1898. [2] Amer. Jour. Med. Sci., Jan. 1893. [3] Arch. of Pediatr., Jan. 1898. [4] Lancet, Feb. 12 and 26, Mar. 12, 1898. [5] Scot. Med. and Surg. Jour., Mar. 1898. [6] Brit. Med. Jour., Mar. 5, 1898. [7] Lancet, Jan. 8, 1898. [8] Med. Week, Aug. 20, 1897. [9] Lancet, Mar. 12, 1898. [10] Lancet, Mar. 19, 1868.

